# Post-COVID Sequelae: From Lung Disease to Long Disease

**DOI:** 10.7759/cureus.35668

**Published:** 2023-03-01

**Authors:** Mohaymin Kadir, Tanjeev Ahmad, Jennifer Bass

**Affiliations:** 1 Department of Internal Medicine, Michigan State University, Lansing, USA; 2 Department of Internal Medicine, Tulane University School of Medicine, New Orleans, USA

**Keywords:** acute asthma, reactive airway diseases, long covid, post-covid sequelae, covid-19

## Abstract

COVID-19 can have both an acute phase and post-acute phase of illness termed post-COVID sequelae, or “long Covid.” In this case, a 66-year-old woman with a past medical history of reactive airway disease was admitted for shortness of breath twice. The first episode occurred in the setting of active COVID-19 infection. However, the second episode took place seven weeks later in the absence of COVID-19 as evidenced by a rapid antigen test. It is unclear why she re-developed shortness of breath after being discharged symptom-free from her initial admission. After treatment with prednisone, albuterol, and ipratropium she experienced symptomatic relief yet again and outpatient pulmonary function testing demonstrated a mildly obstructive pattern reversed with an inhaled bronchodilator. She has remained symptom-free since finishing an outpatient prednisone course. It is possible she developed post-COVID sequelae resembling an acute asthma exacerbation. Though the exact mechanism of post-COVID sequelae is not known, it is thought to be due to a combination of immune activation, dysregulation, and suppression. It is an important presentation for internists to know given the prevalence of COVID-19.

## Introduction

COVID-19 is well known for its acute phase which can vary from a mild to severe presentation. In its mild form a patient may only experience a dry cough and runny nose but in a severe presentation chest pain, fevers, chills, and shortness of breath may be present with associated pneumonia [1]. However, patients who recover from this acute phase may still be at risk for a post-acute phase of COVID-19, post-COVID sequelae also known as “long Covid.” Post-COVID sequelae can occur as a continuation of the acute phase or can present later as a delayed complication of the acute phase. These symptoms can overlap with the acute phase and include fatigue, dyspnea, fevers, cough, myalgias, etc [2]. Though the exact mechanism of this post-acute phenomenon is not completely understood, it is thought to be a result of an overwhelming immune response followed by a potentially prolonged compensatory counterbalancing anti-inflammatory cascade [2]. This immune system activation and suppression can result in a large toll on the body responsible for many symptoms experienced by patients in the post-acute phase. The following case is a patient who had initially experienced the acute phase of COVID-19 only to find herself with the same symptoms, weeks later, in the post-acute phase.

## Case presentation

**Figure 1:**
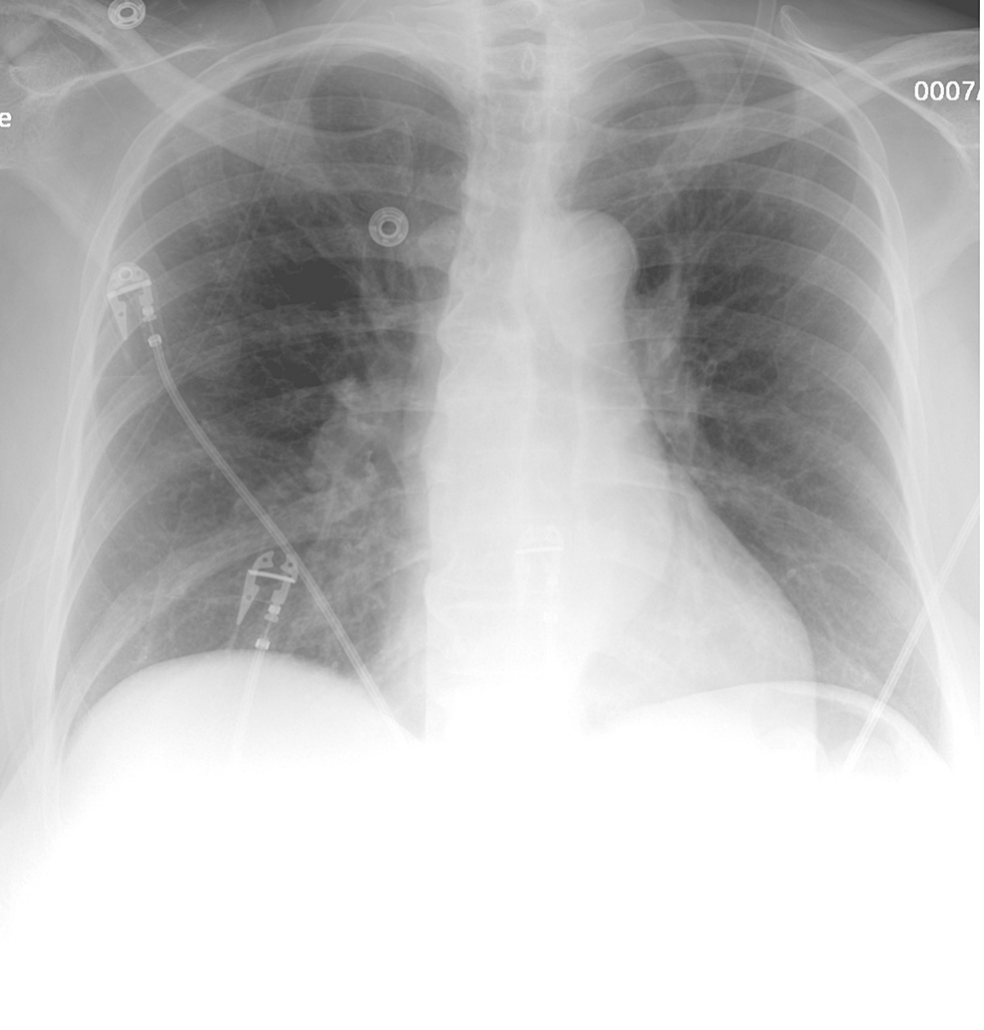
Chest X-ray with no radiographic evidence of an acute cardiopulmonary process

**Figure 2:**
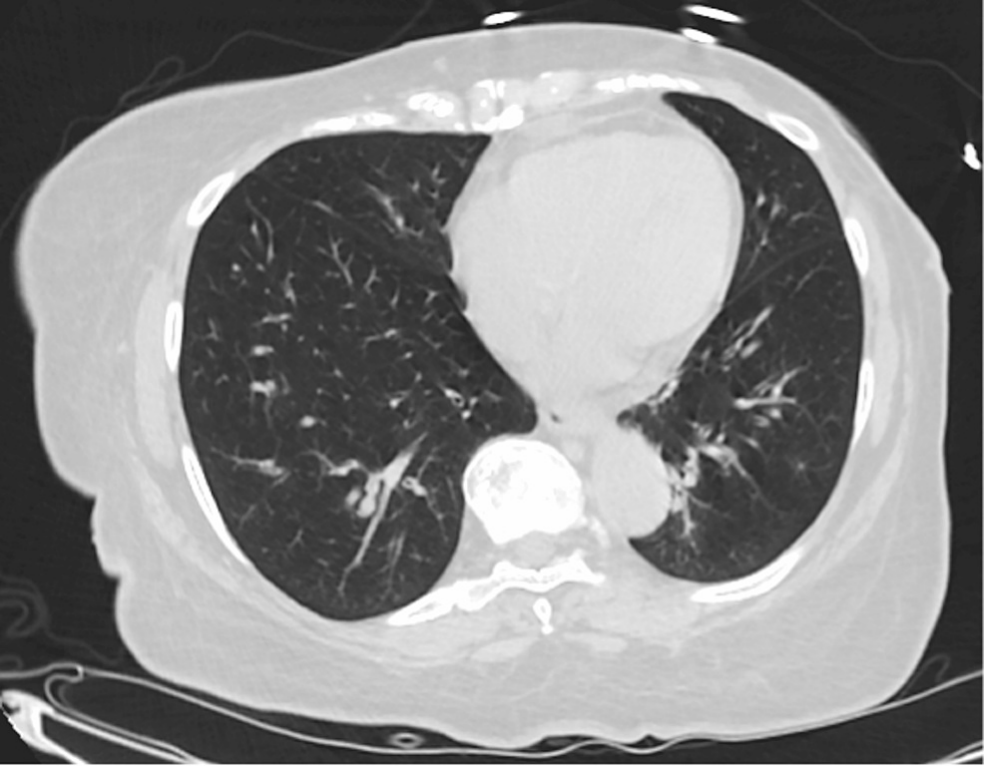
Normal chest CT. No evidence of pulmonary interstitial disease

A 66-year-old woman with a remote history of reactive airway disease presented for a few days of dyspnea. She had been exposed to COVID-19 at a family barbecue a week and a half prior and when she arrived she was positive for COVID-19 and was treated with prednisone and albuterol with immediate improvement and promptly discharged from the ED. Despite being discharged symptom-free initially, she returned short of breath seven weeks later but this time with a negative COVID test. On physical examination, she was tachycardic, tachypneic, and saturating 88% on room air with expiratory wheezes on lung auscultation. She was afebrile. CBC, CMP, and D-dimer were unremarkable. The respiratory panel was negative. A chest X-ray showed no evidence of acute cardiopulmonary disease (Figure 1). High-resolution CT chest showed no infiltrate, effusion, or ground-glass opacities (Figure 2). Given the patient’s remote history of reactive airway disease, treatment was started for asthma exacerbation. She was placed on 4L of oxygen by nasal cannula, and given prednisone, albuterol, and ipratropium. The next day she showed a marked improvement in her heart rate and respiratory status, and was saturating 96-98% on room air. At her pulmonology follow-up appointment, a pulmonary function test (PFT) showed a mildly obstructive pattern reversible with an inhaled bronchodilator. The results are as follows: FEV1/FVC: 70% FEV1: 100 % (2) FVC: 111% (2.8) TLC: 85% (4.4) DLCO: 109%. 

## Discussion

COVID-19 has resulted in a growing population of patients who recover from the acute infection but have the potential to develop long-term sequelae. It is important for internists to recognize post-COVID sequelae, nicknamed “long COVID” [3]. COVID has an acute phase that lasts up to four weeks, and in some patients, has a post-acute phase encompassing a broad range of symptoms that are not explained by an alternative diagnosis. The most frequent symptoms are fatigue, dyspnea, chest pain/tightness, and cough but include many others [3]. At this time there are no widely accepted clinical criteria for the diagnosis of post-COVID sequelae. It is also unclear what triggers post-COVID sequelae, but it is thought to be due to a combination of immune dysregulation, auto-immunity, occult viral persistence, endothelial dysfunction, as well as coagulation activation [4]. Despite the lack of clear diagnostic criteria and because of the novelty of post-COVID sequelae, it is important for hospitalists to suspect it and obtain a comprehensive evaluation of the patient’s COVID history to appropriately evaluate and treat it. Patients with a history of severe COVID infection may be at risk of longer hospitalizations for post-COVID sequelae. Additionally, patients in the post-acute period are at high risk for fibrosis of the lungs regardless of infection severity due to TGF-b upregulation healing lung parenchyma but also proliferating fibroblasts resulting in extracellular matrix accumulation [5]. Finally, though post-COVID sequelae presented solely in the lungs in this case, it has the potential to affect multiple organs.

## Conclusions

In summary, evaluation of a symptomatic patient with prior COVID infection should prompt a comprehensive physical and history with workup as appropriate while maintaining a broad differential. However, post-COVID sequelae should be a part of that differential especially if there is no alternative explanation given the potential for disease progression in the patient. This patient with a history of reactive airway disease may have been predisposed to a post-COVID sequelae resembling an acute asthma exacerbation, but it is unclear what other risk factors may be contributory in the general population.
